# Practice patterns of ureteral access sheath during ureteroscopy for nephrolithiasis: a survey among endourologists worldwide

**DOI:** 10.1186/s12894-019-0489-x

**Published:** 2019-07-04

**Authors:** Dorit Esther Zilberman, Alon Lazarovich, Harry Winkler, Nir Kleinmann

**Affiliations:** Department of Urology , Chaim Sheba Medical Center, Affiliated to Sackler School of Medicine, Tel-Aviv University, Tel-Hashomer, Ramat-Gan, 52621 Tel-Aviv, Israel

**Keywords:** Endourology, Nephrolithiasis, Practice patterns, Ureteral access sheath, Ureteroscopy

## Abstract

**Background:**

The use of ureteral access sheath (UAS) during ureteroscopy is controversial. We aimed to explore practice patterns of UAS during ureteroscopy for nephrolithiasis among endourologists worldwide.

**Methods:**

A 15-question survey was designed using the SurveyMonkey® platform. The questions covered the background and professional experience of the potential respondents, indications for UAS insertion, UAS caliber and possible complications associated with its use.

The questions were anonymously tabulated in order to determine practice patterns of UAS during ureteroscopy for nephrolithiasis among endourologists.

The survey was then distributed via e-mail to all the Endourological Society members.

**Results:**

216 members responded. 99.53% of the respondents practice as endourologists, 63.4% are fellowship trained and 74.4% are at least 6 years post-fellow. 73.2% practice in an academic facility. 77.3% perform at least 100 ureteroscopies annually. 46 and 76% routinely use UAS for the treatment of ureteral and kidney stones, respectively. In both cases, the 12/14 access sheath is the most common. 42% use UAS in primary ureteroscopy. 90.3% believe that a double J stent insertion is not mandatory prior to UAS insertion. 79.1% think the use of UAS does not increase postoperative complications rate, and if the latter does encounter, then most likely it is either a ureteral stricture (93.2%) or pain (48%).

**Conclusions:**

UAS is commonly used by highly skilled endourologists during ureteroscopy. 12/14 UAS is mostly used. Ureteral stricture and post-operative pain are proposed as possible complications following UAS introduction, however pre-stenting is not mandatory as overall low complication rate is expected.

## Background

The ureteral access sheath (UAS) was first introduced in 1974 [1]. The stiff and hollow tube that literally paved the road to the upper urinary tract had carried the promise to smoother and easier surgical manipulations in a territory considered by then as difficult to inspect.

Despite its tremendous potential, an ongoing controversy still exists in the literature as regards to UAS safety and necessity during ureteroscopy.

The supporters, on one hand, point out its advantages, such as: better visibility [2], increased stone free rates (SFR) [3], decreased likelihood of infection [4] and bleeding [5], decreased intra-pelvic irrigation pressure [6–8], decreased wear of the flexible ureteroscope [2, 9] and shortening of total operative time [10].

The critics, on the other hand, emphasize traumatic insertion and the pressure the UAS poses over the ureteric tissue that in turn may result in ischemic injury and ureteric strictures [11–14].

Moreover, per their doctrine, UAS has no influence neither on SFR [4, 15] nor on total operative time [4]. However, it might contribute to higher rates of post-operative complications [16] and longer hospital stay [4].

In light of this controversy we sought to explore practice patterns of ureteral access sheath during ureteroscopy for nephrolithiasis among endourologists worldwide.

## Methods

The current urological literature regarding UAS utilization was reviewed.

A 15-question survey was then designed using the SurveyMonkey® platform to cover practice patterns and known controversies which currently exist as regards to the application of UAS during ureteroscopy.

The questionnaire entitled “ Practice Patterns Of Ureteral Access Sheath During Ureteroscopy For Nephrolithiasis”, consisted of multi-choice answers varied between 2 (i.e: yes/no) to 5 answers options. In one question the respondents were allowed to check more than one box.

The questions covered the background and professional experience of the potential respondents, indications for UAS insertion, UAS caliber and possible complications associated with its insertion.

The questions were anonymously tabulated in order to determine practice patterns of UAS during ureteroscopy among endo-urologists.

The survey was subsequently distributed with the assistance of the Endourological Society office via e-mail to all the Endourological Society members.

Descriptive statistics and survey analysis were executed using the SurveyMonkey® platform.

If a respondent skipped a question, the response for that question was not included in the analysis.

## Results

Out of estimated 2000 registered Endourological Society members who received the questionnaire, 216 responded.

Questions and responses are detailed in Table 1.

**Table 1:** Survey’s questions and responses

Q1: Do you practice as an Endourologist?		
Answer Choices	Responses	
Yes	99.53%	213
No	0.47%	1
Total		214
Q2: Are you a fellowship-trained Endourologist?		
Answer Choices	Responses	
Yes	63.43%	137
No	36.57%	79
Total		216
Q3: How many years have passed since you graduated from your fellowship?		
Answer Choices	Responses	
0–5	25.59%	54
6–10	17.54%	37
> 10	56.87%	120
Total		211
Q4: Where do you practice?		
Answer Choices	Responses	
University hospital	54.46%	116
Private practice	26.76%	57
Combination university hospital and private practice	18.78%	40
Total		213
Q5: What is your yearly volume of ureteroscopy?		
Answer Choices	Responses	
< 100	22.69%	49
100–199	33.33%	72
200–299	23.61%	51
300–400	12.96%	28
> 400	7.41%	16
Total		216
Q6: Do you routinely use ureteral access sheath (UAS) for the treatment of ureteral stones?		
Answer Choices	Responses	
Yes	45.83%	99
No	54.17%	117
Total		216
Q7: In what percentage of cases do you use UAS?		
Answer Choices	Responses	
0–20%	4.55%	5
20–40%	6.36%	7
40–60%	26.36%	29
60–80%	30.91%	34
80–100%	31.82%	35
Total		110
Q8: What is the UAS size that you commonly use?		
Answer Choices	Responses	
9.5/11.5 F	11.82%	13
10/12 F	26.36%	29
12/14 F	44.55%	49
14/16 F	2.73%	3
Other	14.55%	16
Total		110
Q9: Do you routinely use UAS for the treatment of kidney stones?		
Answer Choices	Responses	
Yes	75.71%	159
No	24.29%	51
Total		210
Q10: In what percentage of cases do you use UAS?		
Answer Choices	Responses	
0–20%	5.03%	8
20–40%	6.92%	11
40–60%	15.72%	25
60–80%	25.79%	41
80–100%	46.54%	74
Total		159
Q11: What is the UAS size that you commonly use?		
Answer Choices	Responses	
9.5/11.5 F	14.01%	22
10/12 F	24.2%	38
12/14 F	45.86%	72
14/16 F	4.46%	7
Other	11.46%	18
Total		157
Q12: Do you use UAS in primary ureteroscopy?		
Answer Choices	Responses	
Yes	42.03%	87
No	57.97%	120
Total		207
Q13: Is a double J stent insertion mandatory prior to UAS insertion?		
Answer Choices	Responses	
Yes	9.66%	20
No	90.34%	187
Total		207
Q14: Do you think the use of UAS increases complications rate?		
Answer Choices	Responses	
Yes	20.87%	43
No	79.13%	163
Total		206
Q15: Which of the following? (you can choose more than one)		
Answer Choices	Responses	
Ureteral strictures	93.18%	41
Hydronephrosis	13.64%	6
Infection	6.82%	3
Pain	47.73%	21
Total respondents: 44		

99.53% of the respondents practice as endourologists, almost two thirds of whom are fellowship trained endourologists and 74.4% are at least 6 years post-fellow. 73.2% practice in an academic facility, either in a university hospital or both university hospital and private practice.

77.3% perform at least 100 ureteroscopies annually.

Almost 46% routinely use UAS for the treatment of ureteral stone, and the rate further increases into 75.7% when it comes to treating kidney stones.

In both cases, the 12/14 access sheath is the most common (44.5 and 46%, respectively).

42% use UAS in primary ureteroscopy. 90.3% believe that a double J stent insertion is not mandatory prior to UAS insertion.

79.1% believe the application of UAS does not increase postoperative complications rate, and if the latter does occur, then most likely it is either a ureteral stricture (93.2%) or pain (48%).

## Discussion

In 2007, Auge et al. distributed a 26-question survey to 570 urologists worldwide [17]. The aim of the survey was to assess practice patterns of ureteral stenting after routine ureteroscopic stone surgery.

Of the 173 respondents, only 21% dilated the ureteral orifice 90% of the time, and only 34.5% of those who did so used an access sheath either alone or in combination with ureteral balloon.

Clearly, ever since that time the endourology has become an established sub specialty, and the penetrance of designated devices has increased accordingly.

The present survey reflects highly skilled and trained endourologists:

99% of the respondents define themselves as endourologists; 63% graduated an established endourology fellowship program; most respondents have at least 6 years post-fellow seniority; 73% are affiliated to academic centers and 77% maintain high annual volume of at least 100 procedures.

This highly skilled study population uses access sheath in higher percentages than described earlier: 46 and 76% use access sheath for ureteral stones and kidney stones treatment, respectively, rates that are even higher than the 67% reported elsewhere [4].

In a study evaluating different UASs available in the international market and their compatibility with different available flexible ureteroscopes, the 12/14 access sheath was found to be the device that accepts all scopes [18].

Another study assumed that this sheath decreases the likelihood of infection compared to smaller caliber UAS [5].

This may explain the popularity of the 12/14 UAS in the present survey, as 44.5 and 46% of the endourologists use it for the treatment of ureteral and kidney stones, respectively.

Apparently, the rate of UAS introduction in ureteral stones treatment is relatively low (42%). The present survey did not split stone location in accordance with different portions of the ureter. By doing so, we would have probably been seeing higher percentages of UAS introduction mainly for proximal ureteral stones treatment.

Yet, the relatively low popularity of the UAS for ureteral stone treatment can be explained by the small caliber semi-rigid ureteroscopes which currently exist in the market, that in turn enable atraumatic exploration of the ureter even in its upper portions.

The latter may also explain why 90.3% of the survey respondents believe pre-stenting prior to UAS introduction is not mandatory, a finding that stands in line with another study observation [4], whereby of the 1494 patients who underwent UAS introduction, only 36% were pre-stented.

Both the present study and Traxer et al. study [4] contradict other studies which support pre-stenting prior to ureteroscopy and UAS insertion in order to decrease the likelihood of ureteral injury [12] and UAS insertion failure rates [19–21].

Overall, 79% of the survey respondents believe UAS insertion does not increase post operative complications rate. When a complication arises, the highest likelihood it is of a stricture kind (93%).

Delvecchio et al. [14] reviewed ureteral stricture rates following UAS insertion in 62 patients with at least 3 months post-operative follow up. In this study, a 12/14 UAS was applied in 79% of the cases. Only one case demonstrated a UPJ stricture that was not attributed to the UAS. Their conclusion was, therefore, that large caliber UAS should be used with caution especially when long operative time and ureteric ischemic injury are expected.

Risk factors for ureteral wall injuries resulting from insertion of UAS during retrograde intrarenal surgery, as declared by one study [12] are as follows: older age, male gender, absent ureteral double J stenting before procedure.

Another potentially commom complication the respondents mentioned in the present survey was pain (48%).

Lildal et al. [11], in a benchtop trial, inserted UASs in 44 porcine ureters for 2 min on one side and for 2 h on the contra-lateral side.

The ureters were excised and tissue samples from the proximal and the distal ureters were detected for mRNA of the inflammatory mediators COX-2 and TNF-α.

After UAS insertion for 2 min, mRNA expression levels of COX-2 and TNF-α in the distal ureters increased 6.5 and 8 fold, respectively, and after 2 h the levels further increased to as high as 9 and 9.5 fold, respectively.

Expression levels were significantly higher in the distal than in the proximal portion of the UAS treated ureters, and significantly higher in UAS treated ureters compared to controls.

These findings may explain the relatively high percentage of supposedly pain in the survey, which presumably reflects individual observation and personal experience of each respondent with his or her own cases.

Over the past 15 years, the endourological field has been evidencing technological advancements that have led to the introduction of numerous devices with various physical properties, that have made the lives of many urologists worldwide easier.

The UAS, from this standpoint, is of no exception.

The present survey does not come up with substantial recommendations as regards to when and where to use UAS.

It does not define practice patterns of UAS in different parts of the world either.

Another limitations that should be mentioned are the relatively low response rate (10.8%) as well as unknown number of manufacturers and substances involved in UASs’ production.

Notwithstanding, the additional scientific value of this survey is the contemporary perspective it provides as regards to UAS introduction among high number (the highest so far) of highly skilled endourologists.

Moreover, it emphasizes the significant role UAS plays in the urologists’ current armamentarium.

## Conclusions

UAS is commonly used by highly skilled endourologists during ureteroscopy. 12/14 UAS is mostly used. Ureteral stricture and post-operative pain are proposed as possible complications following UAS introduction, however pre stenting is not mandatory as an overall low complication rate is expected .

## Data Availability

All data generated or analyzed during this study are included in this published article.

## Abbreviations

SFR: Stone Free Rates
UAS: Ureteral Access Sheath
URS: Ureteroscopy
